# Supplementary material for: The causal relationship between the human gut microbiota and pyogenic arthritis: a Mendelian randomization study
Source: Front Cell Infect Microbiol. 2024 Nov 26;14:1452480. doi: 10.3389/fcimb.2024.1452480 (PMC11629706; doi:10.3389/fcimb.2024.1452480)
Supplement: Supplementary file 1 [file DataSheet1.zip › mendelian test/ebi-a-GCST90027689.csv_forest.pdf]

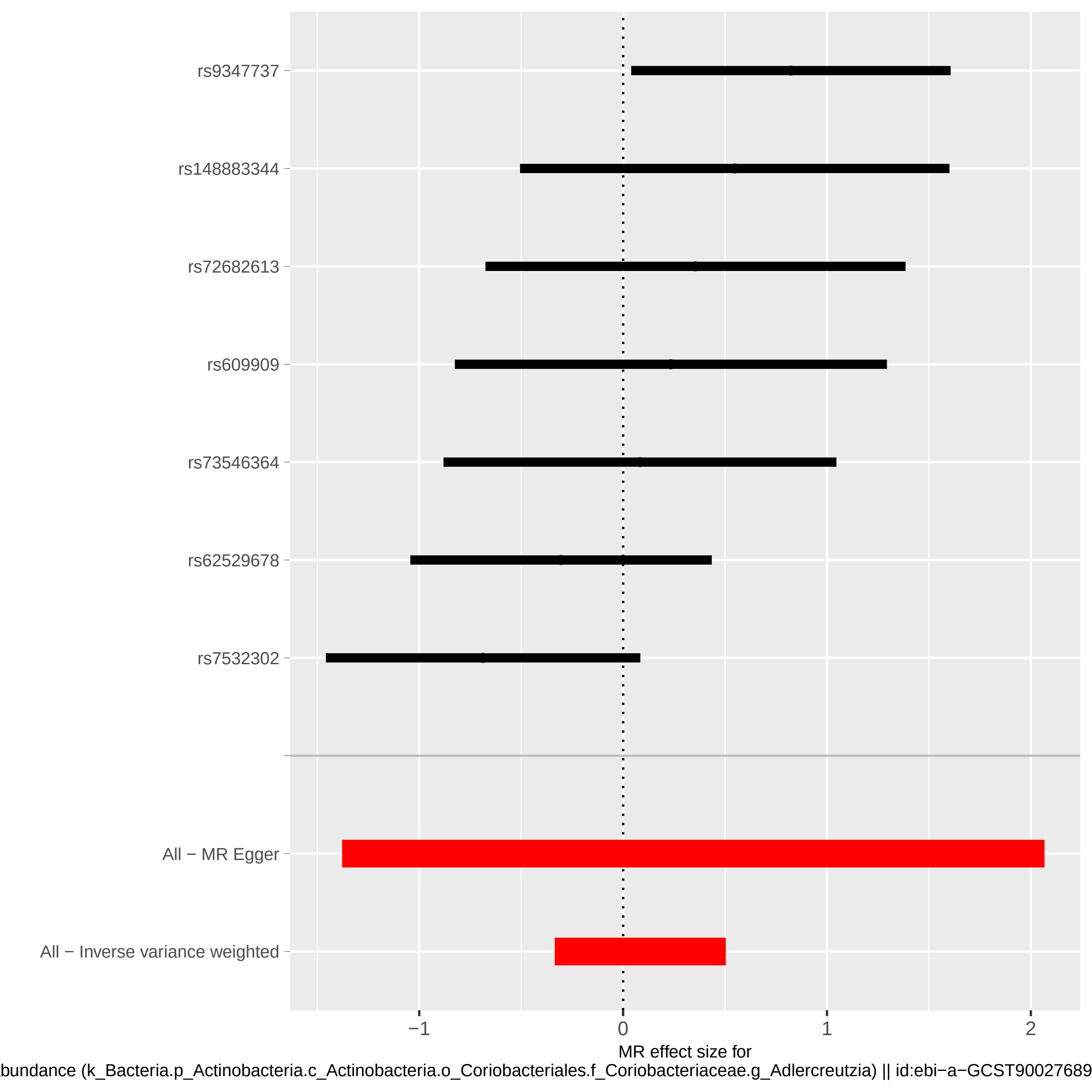
rs9347737
rs148883344
rs72682613
rs609909
rs73546364
rs62529678
rs7532302
All − MR Egger
All − Inverse variance weighted
−1
0
1
2
MR effect size for
bundance (k_Bacteria.p_Actinobacteria.c_Actinobacteria.o_Coriobacteriales.f_Coriobacteriaceae.g_Adlercreutzia) || id:ebi−a−GCST90027689
